# Development and Validation of a Web-Based Dynamic Nomogram to Improve the Diagnostic Performance of Subscapularis Tendon Tear

**DOI:** 10.3389/fsurg.2022.874800

**Published:** 2022-05-31

**Authors:** Wennan Xu, Zitian Zheng, Qingyun Xue

**Affiliations:** ^1^ Orthopaedics Department, Beijing Hospital, National Center of Gerontology, Institute of Geriatric Medicine, Chinese Academy of Medical Sciences, Beijing, China; ^2^ Graduate School of Peking Union Medical College, Beijing, China

**Keywords:** subscapularis, nomogram, dynamic, prediction, tear

## Abstract

**Background:**

There are still some challenges in diagnosing subscapularis (SSC) tendon tears as accurately as posterosuperior rotator cuff tears on magnetic resonance imaging (MRI). The omission of SSC tendon tears can lead to muscle atrophy, fatty infiltration and increased tear accompanied by aggravated shoulder pain and loss of function. An effective noninvasive evaluation tool will be beneficial to early identification and intervention. The study aims to identify sensitive predictors associated with SSC tendon tears and develop a dynamic nomogram to improve diagnostic performance.

**Methods:**

From July 2016 to October 2021, 528 consecutive cases of patients who underwent shoulder arthroscopic surgery with preoperative shoulder MRI were retrospectively analyzed. The least absolute shrinkage and selection operator (LASSO) method was used to identify the sensitive factors associated with SSC tendon tears, which were then incorporated into the nomogram. The prediction performance of the nomogram was evaluated by concordance index (C index) and calibrated with 1,000 bootstrap samples combined with external validation of another cohort.

**Results:**

The LASSO method showed that six items including coracohumeral distance (oblique sagittal plane), effusion (Y-face), effusion (subcoracoid), malposition of the long head tendon of the biceps, multiple posterosuperior rotator cuff tears, and considering SSC tendon tears on MRI (based on direct signs) were determined as sensitive predictors. The nomogram achieved a good C index of 0.878 (95% CI, 0.839–0.918) with a good agreement on the risk estimation of calibration plots. The areas under the receiver operator characteristic (ROC) curves of the two methods showed that dynamic nomograms had better prediction performance than MRI diagnosis based on direct signs (training set 0.878 vs. 0.707, validation set 0.890 vs. 0.704).

**Conclusion:**

The study identified sensitive predictors associated with SSC tendon tears and first developed a web-based dynamic nomogram as a good supplementary evaluation tool for imaging diagnosis that could provide an individualized risk estimate with superior prediction performance, even in patients with small or partial tears.

## Introduction

As the largest and most powerful tendon of the rotator cuff, SSC plays an important role in maintaining the stability of the glenohumeral joint and internal rotation of the shoulder joint. The incidence of SSC tendon tears has been reported in 12–50% of all patients undergoing arthroscopy (1–3). Lesions involving SSC can lead to long-term shoulder pain and progressive loss of function. Although the diagnostic performance of magnetic resonance imaging (MRI) in supraspinatus and infraspinatus tendon tears is excellent, it has been challenging to find MRI as an effective diagnostic tool for SSC tendon tears (4–6). Especially, the smaller and partial-thickness tears could directly decrease the diagnostic accuracy of MRI (7–9). Considering the SSC tendon tear usually begins at the upper part of the tendon insertion, most of which occurred in the superior one-third of the tendon insertion (10), the presence of a partial volume effect will also make it difficult to visualize the lesions in the anterosuperior region (11).

Although a good clinical outcome was obtained after arthroscopic repair of SSC tendon tears (12), most tears were mainly identified during an arthroscopic examination of other shoulder injuries. The preoperative omission of these lesions can lead to muscle atrophy, fatty infiltration, and increased tearing accompanied by aggravated shoulder pain and loss of function (13). Because of the difference in clinical experience, the accuracy of diagnosis varies among clinicians (14). An effective risk prediction tool will assist clinicians in early identifying such injuries. Although researchers have reported some factors associated with SSC tendon tears (15–18), it was unclear which were the pivotal predictors and the diagnostic value of them in predicting SSC tendon tears. It is crucial to improve the diagnostic accuracy of SSC tendon tears, and we hope to develop a reliable prediction system to convert this speculative experience to scientific risk estimation to assist the early diagnosis and intervention.

Of all the available models, a nomogram can provide an evidence-based, individualized, and highly accurate risk estimation. This study hypothesized that this new predictive system could provide superior diagnostic performance in identifying SSC tendon tears.

## Materials and Methods

### Patient Cohort

We retrospectively analyzed data of patients who received shoulder arthroscopic procedures at our medical institution from July 2016 to October 2021 and identified 528 consecutive cases with complete data of shoulder MRI for inclusion in the study. This study was approved by the ethics commissions of our hospital. The requirement for patient consent was waived by the review board because of the retrospective nature of the study. Three hundred and sixty-two patients (243 females and 119 males, mean age: 60.56 years) who underwent shoulder arthroscopy from July 2016 to July 2019 were included in the training data set, and 166 patients (109 females and 57 males, mean age: 60.85 years) who received shoulder arthroscopy from August 2019 to October 2021 were included in the validation data set. Inclusion criteria were patients who underwent shoulder arthroscopic procedures with preoperative shoulder MRI. Patients combined with rheumatic immune diseases, infections, tumors, and revision surgery of the shoulder were excluded.

Demographic data including gender, age, dominant hand, cause of injury, comorbidities, cigarette smoking, and alcohol consumption were collected. The severity of an SSC tendon tear was determined arthroscopically according to the Lafosse classification (19). Yoo et al. divided the SSC tendon footprint into four distinct facets (facet 1–4) through a cadaveric observational study (10). They provided the facet’s dimensions and surface area, convenient for arthroscopic measurements. We measured SSC tear size during the arthroscopy by a numeric probe with a scale of 1 mm to describe the classification (Lafosse I–V). Patients with arthroscopically determined SSC tendon tears were included in the SSC tear group, while others with intact SSC were enrolled in the non-SSC tear group. Ultimately, 86 patients (mean age: 63.12 years) and 276 patients (mean age: 59.76 years) were enrolled in the SSC tear group and non-SSC tear group, respectively. According to Lafosse’s classification, SSC tendon tears of the training cohort occurred in the upper third in 51 patients (Lafosse I/II type), and the remaining 35 patients had SSC tendon tears that exceeded the upper third (Lafosse III–V type). In the validation cohort, 35 patients combined with upper third SSC tendon tears, and 18 patients had tears that exceeded the upper third of SSC tendon (Lafosse III–V type). A detailed description of demographic data is reported in Table 1.

**Table 1:** Clinical and imaging features of SSC tears in the development and validation cohorts.

Variable	Development cohort (*n* = 362)		*p* Value	Validation cohort (*n* = 166)		*p* Value
	SSC tear (*n* = 86)	Non-SSC tear (*n* = 276)		SSC tear (*n* = 53)	Non-SSC tear (*n* = 113)	
Age, year	63.12 ± 8.82	59.76 ± 9.42	0.003	62.87 ± 8.39	59.90 ± 9.15	0.047
Gender						
Male	27	92	0.738	17	40	0.674
Female	59	184		36	73	
Cause						
Degenerative	44	131	0.549	33	56	0.126
Traumatic	42	145		20	57	
Dominant hand						
Yes	53	155	0.370	31	65	0.906
No	33	121		22	48	
CHD (axial), mm	7.88 ± 2.11	9.28 ± 2.46	<0.001	8.23 ± 2.02	9.69 ± 2.49	<0.001
CHD (oblique sagittal), mm	8.09 ± 2.02	9.73 ± 2.60	<0.001	8.23 ± 1.70	10.29 ± 2.67	<0.001
CO, mm	16.10 ± 3.92	15.84 ± 3.94	0.587	15.54 ± 4.01	15.38 ± 4.29	0.812
CHI	0.33 ± 0.08	0.33 ± 0.08	0.334	0.32 ± 0.08	0.32 ± 0.09	0.522
Effusion (en-face)						
Yes	73	191	0.004	44	71	0.009
No	13	85		9	42	
Fluid area ratio (en-face), ratio >0.5						
Yes	14	12	<0.001	11	1	<0.001
No	72	264		42	112	
Effusion (Y-face)						
Yes	28	23	<0.001	12	5	<0.001
No	58	253		41	108	
Effusion (Coronal)						
Yes	60	140	0.002	36	43	<0.001
No	26	136		17	70	
Atrophy (en-face)						
Grade I	61	256	<0.001	41	103	0.015
Grade II/III	25	20		12	10	
Atrophy (Y-face)						
Grade I	49	218	<0.001	34	83	0.221
Grade II/III	37	58		19	30	
Effusion (subcoracoid)						
Yes	53	73	<0.001	31	24	<0.001
No	33	203		22	89	
Lesser tuberosity cyst, number ≥1						
Yes	29	71	0.148	18	35	0.700
No	57	205		35	78	
Lesser tuberosity cyst, diameter r≥5 (mm)						
Yes	9	13	0.051	5	3	0.057
No	77	263		48	110	
LHB dislocation/subluxation						
Yes	33	24	<0.001	17	14	0.002
No	53	252		36	99	
Patte						
Normal/grade I	55	246	<0.001	37	104	<0.001
Grade II/III	31	30		16	9	
Classification						
Non-full-thickness tear	38	219	<0.001	25	96	<0.001
Full-thickness tear	48	57		28	17	
Number of PS rotator cuff tears						
≤1	42	248	<0.001	27	105	<0.001
≥2	44	28		26	8	
Hypertension						
Yes	39	96	0.077	24	37	0.118
No	47	180		29	76	
Diabetes						
Yes	26	49	0.013	18	22	0.042
No	60	227		35	91	
Coronary heart disease						
Yes	21	28	0.001	13	8	0.002
No	65	248		40	105	
Cerebral infarction						
Yes	6	12	0.327	4	4	0.261
No	80	264		49	109	
Smoking history						
Yes	7	34	0.286	5	14	0.577
No	79	242		48	99	
Drinking history						
Yes	7	17	0.519	5	6	0.319
No	79	259		48	107	
SSC tear (direct signs on MRI)						
Yes	49	43	<0.001	31	20	<0.001
No	37	233		22	93	

All patients who underwent shoulder arthroscopic surgery were placed in the beach chair position with general anesthesia, and all of the procedures were performed by two comparably senior shoulder surgeons. The main surgical procedures included (1) arthroscopic exploration and debridement, (2) subacromial decompression, (3) adhesion release, (4) rotator cuff repair, (5) fixation of the labrum, (6) tenotomy or tenodesis of the long head of the biceps, and (7) debridement of calcific tendinitis. If necessary, all patients were instructed to use a shoulder abduction brace immediately with standardized rehabilitation protocol postoperatively. Rehabilitation training was conducted under the guidance of professional rehabilitation physicians.

### MRI Image Evaluation and Definition

All patients included in the study received the standard shoulder MRI protocol. T1-weighted, T2-weighted fast-spin-echo, and fat-suppressed gradient echo and proton density-weighted (PDW) images (thickness = 4/5 mm) were performed using a conventional 3.0-T MRI scanner (Siemens Medical Systems). The scanning direction included axial, oblique coronal (parallel to the long axis of the supraspinatus), and oblique sagittal plane (perpendicular to the long axis of the supraspinatus). The field of view (FOV = 160 mm) was centered on the humeral head with the affected upper limb in a neutral position (hands were placed on the sides of the body, with palms upward). None of the patients received magnetic resonance arthrography (MRA). According to previous reports (17, 20–24), clinical importance, and clinical experience, the study included 17 imaging features to evaluate their association with SSC tendon tears (Table 1). Two previously trained orthopedists evaluated these characteristics. The consensus was reached after deliberation, and the mean value of variables was obtained with twice repeated measurements. The imaging characteristics are hereinafter described in detail and reported in Table 1.

**Figure 1:**
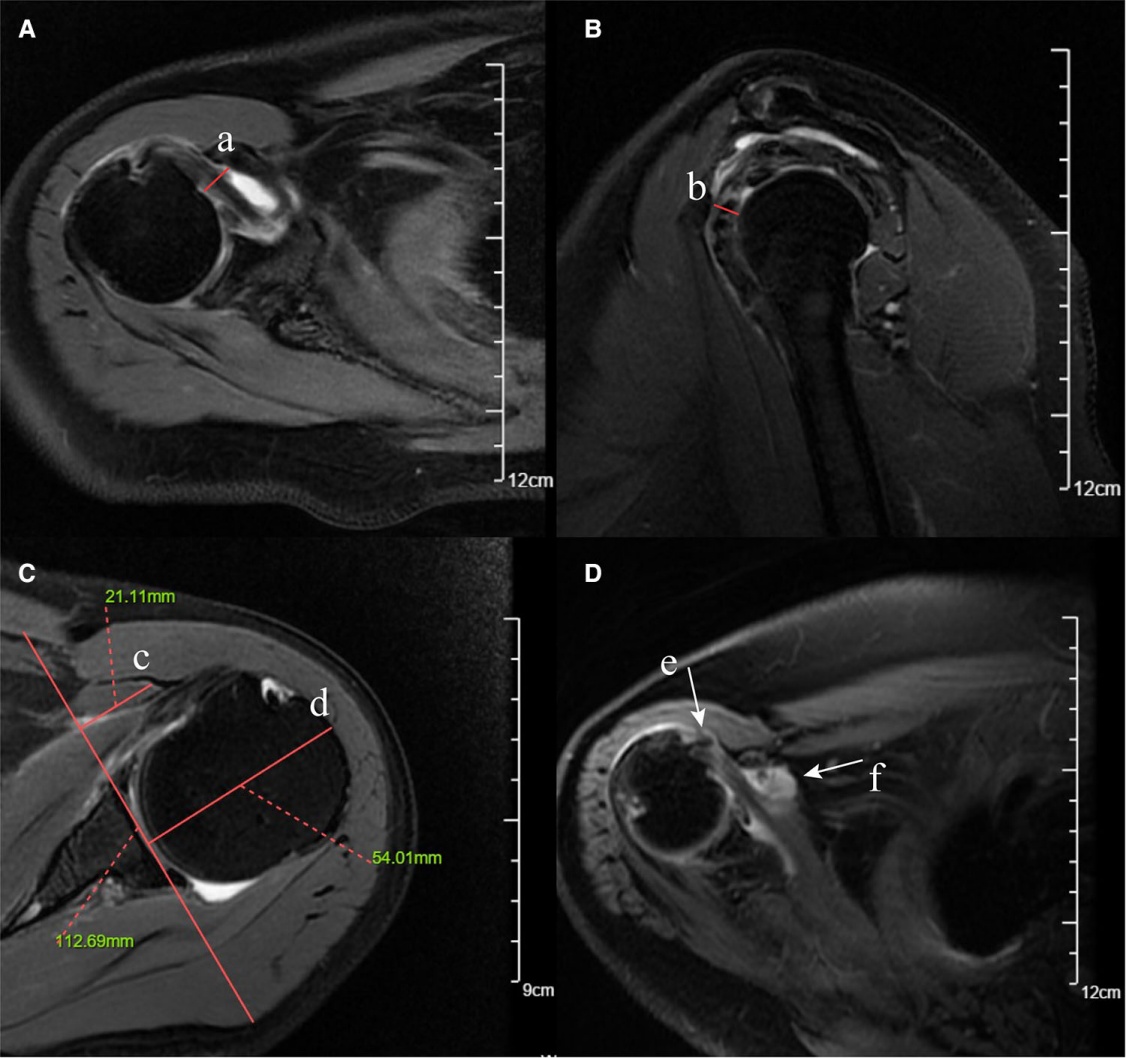
Fat-suppressed T2-weighted MRI images of coracohumeral distance (CHD), coracoid overlap (CO), coracohumeral index (CHI), LHB malposition, and subcoracoid effusion. (**A**) CHD measured on the axial plane (red solid line a). (**B**) CHD measured on the oblique sagittal plane (red solid line b). (**C**) CO (red solid line c) and CHI (red solid line c/ red solid line d, 21.11 mm/54.01 mm). (**D**) LHB malposition on the axial plane (white arrow e). (**E**) Subcoracoid effusion on the axial plane (white arrow f).

Coracohumeral distance (CHD) was defined as the minimal distance between the humeral cortex and the coracoid cortex (20). According to different measurement planes, this parameter could be measured on axial and oblique sagittal planes, respectively (Figure 1). The coracoid overlap (CO) represented the distance from the glenoid to the tip of the coracoid, which was also measured on the axial plane (20) (Figure 1). According to the description of Zhang et al. (21), we calculated the relative ratio of the coracoid length and humeral head diameter as coracohumeral index (CHI) on the axial plane (Figure 1).

**Figure 2:**
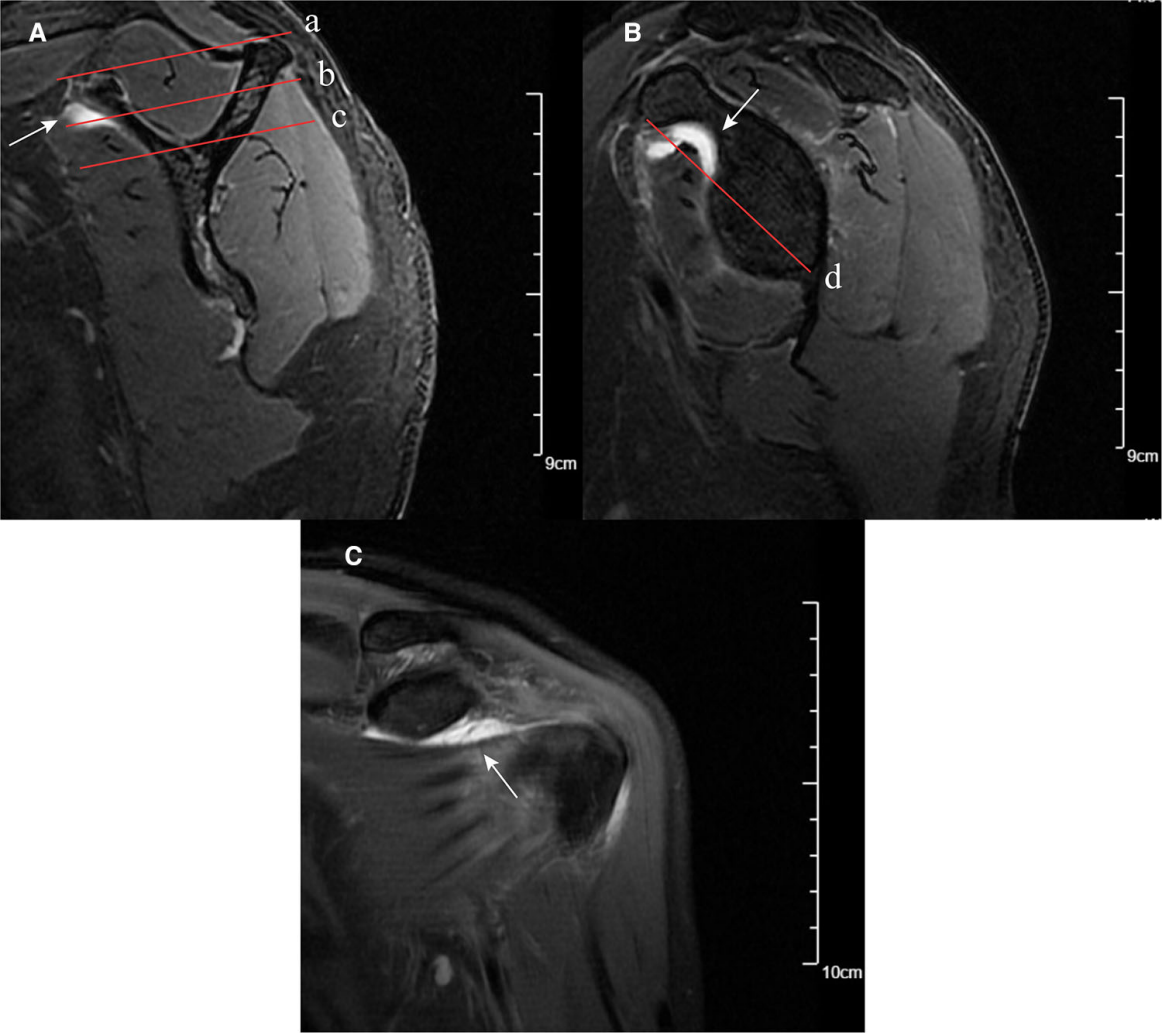
MRI images of subscapular muscle atrophy, effusion, and fluid area ratio (en-face). (**A**) SSC muscle atrophy classification on the Y-face plane. Parallel line intercepting supraspinatus fossa opening (red solid line a) and parallel line (red solid line b) bisecting the perpendicular distance between line (red solid line a) and line (red solid line c). Based on these lines, the SSC position was classified: between lines (a) and (b) was classified as grade (I), between lines (b) and (c) was classified as grade (II), and below line (c) was classified as grade (III). Effusion on Y-face plane (white arrow). (**B**) En-face plane showing the base to-tip line (BTL): the inferior pole of glenoid to coracoid tip on en-face (red solid line d). Using BTL, muscle atrophy of SSC is classified as grade I (tendon and muscle exist above the BTL), grade II (only tendon exists above the BTL), and grade III (tendon and muscle exist below the BTL). The fluid area ratio was the ratio of the effusion area to the area surrounded by the coracoid process, glenoid, and BTL in the en-face. Effusion on en-face plane (white arrow). (**C**) Effusion on the coronal plane (white arrow).

Shim et al. (23) introduced two selected planes (the en-face and Y-face) of the sagittal–oblique plane on MRI for SSC tendon tear measurements. The en-face plane was the image in which the glenoid was the largest observed and the base of the coracoid process was in contact with the glenoid, and the Y-face plane was the first image medial to the glenoid where the scapular spine was in contact with the scapular body (Figure 2). We used these two planes to evaluate the subscapular muscle atrophy and effusion. In the en-face and Y-face, subscapular muscle atrophy can be classified as grades I, II, and III according to the extent of atrophy; the higher the grade, the more severe the atrophy. A detailed description of subscapular muscle atrophy grading is presented in Figure 2. In addition, we described a new index, namely, fluid area ratio, to evaluate further the role of effusion in the en-face plane. According to the base-to-tip line (BTL), we defined the fluid area ratio (en-face) as the ratio of the effusion area to the area surrounded by the coracoid process, glenoid, and BTL in the en-face (Figure 2). To facilitate the measurement of the fluid area ratio (en-face) on MRI, the fluid area ratio was categorized into ratios >0.5 and <0.5.

Given the anatomical proximity of the posterosuperior (supraspinatus, infraspinatus, and teres minor) rotator cuff to the SSC, the retraction and extent of posterosuperior (PS) rotator cuff tendon tear might be associated with SSC tendon tears. The retraction of the PS rotator cuff was graded according to the Patte classification (grades I–III) (23, 25). We evaluated the severity of PS rotator cuff tear according to the thickness (full-thickness vs. nonfull-thickness) and the number of tendon tears (normal/single versus multiple). Yoon et al. demonstrated that malposition (subluxation/dislocation) of the long head tendon of the biceps (LHB) on MRI was associated with a concurrent SSC full-thickness tear (17), and we evaluated the LHB malposition on the axial plane (Figure 1). Mostly, the greater tubercle cysts were assessed to be related to supraspinatus tears (26, 27); some scholars speculated that the lesser tuberosity cyst (LTC) and subcoracoid effusion may be related to SSC tendon tears (18, 24, 25). This study evaluated LTC on the fat-suppressed T2-weighted axial plane to determine the predictive value.

Two researchers evaluated clinical characteristics and indirect imaging features. The diagnosis on MRI according to direct signs (morphology and signal of the tendon) was defined as MRI diagnosis (direct signs), which was evaluated by the musculoskeletal radiologist and another orthopaedist. All of the above investigators were blinded to the grouping of the participant. The MRI diagnosis (direct signs) was divided into two conditions, namely, considering SSC tendon tears or considering non-SSC tendon tears on MRI, which was also regarded as a candidate variable for evaluation. All suspicious diagnoses based on direct signs were considered to be SSC tendon tears.

### Statistical Analysis

Categorical variables were reported as whole numbers, and continuous variables were expressed as means ± standard deviation. The significance of continuous variables was assessed by student’s t-test or nonparametric tests. Chi-square tests or Fishers’ exact test was used in the analysis of categorical variables. Variables associated with SSC tears at a significant level (*p* < 0.1) were candidates for the least absolute shrinkage and selection operator (LASSO) method and 10-fold cross-validation, which is suitable for the regression of high-dimensional data (28). LASSO regression with 10-fold cross-validation was utilized by package “glmnet” in R (version 4.0), and statistical analysis was performed using SPSS (IBM SPSS 25.0).

According to the results of the LASSO method, the “rms,” “DynNom,” and “shiny” packages of the R language version 4.0 (http://www.r-project.org/) were used to construct a web-based dynamic nomogram. The predictive performance of the nomogram was evaluated by the concordance index (C index) and calibrated with 1,000 bootstrap samples. The AUC, specificity, sensitivity, likelihood ratios, and predictive values were calculated to evaluate the diagnostic performance of dynamic nomogram and MRI. Decision curve analysis (DCA) was applied to evaluate the feasibility of this prediction model and MRI.

## Results

### Evaluation and Screening of Predictors

**Figure 3:**
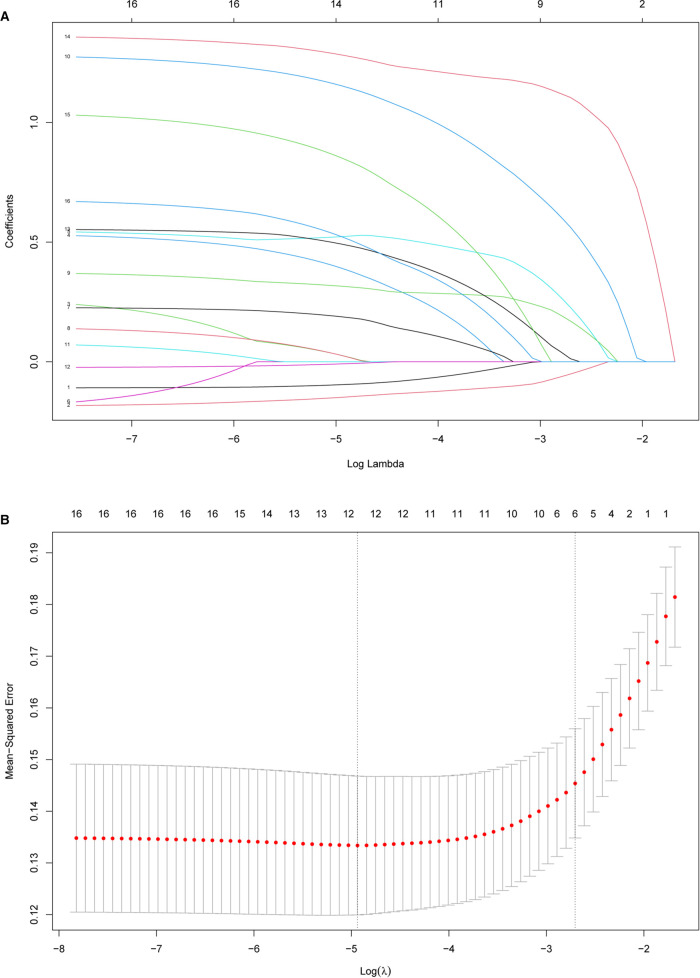
Feature selection using the least absolute shrinkage and selection operator (LASSO) method and cross-validation. (**A**) Extracted features were reduced via the LASSO method and correlation matrix selection. (**B**) Tenfold cross-validation was used to obtain the best lambda value. The minimum log(lambda) value was about −4.9. The vertical line of one minimal standard error was drawn at the right side of the minimum log(lambda), where optimal lambda resulted in six items with nonzero coefficients.

Table 1 presents the clinical and imaging characteristics of patients in the training data set and validation data set. Based on clinical importance, scientific knowledge, and previous reports, a total of 27 variables were selected as candidates. In univariable analysis, 19 items were selected as candidates associated with SSC tendon tears with a *p*-value <0.1. Subsequently, the LASSO method combined with 10-fold cross-validation was performed to reduce the dimensionality of the model and determine the final factors with the most predictive power. The result suggested that decreased CHD (oblique sagittal plane), effusion (Y-face), subcoracoid effusion, LHB malposition, multiple PS rotator cuff tears, and considering SSC tendon tears on MRI (direct signs) were highly associated with SSC tendon tears (Figure 3).

### Development and Evaluation of Nomogram

**Figure 4:**
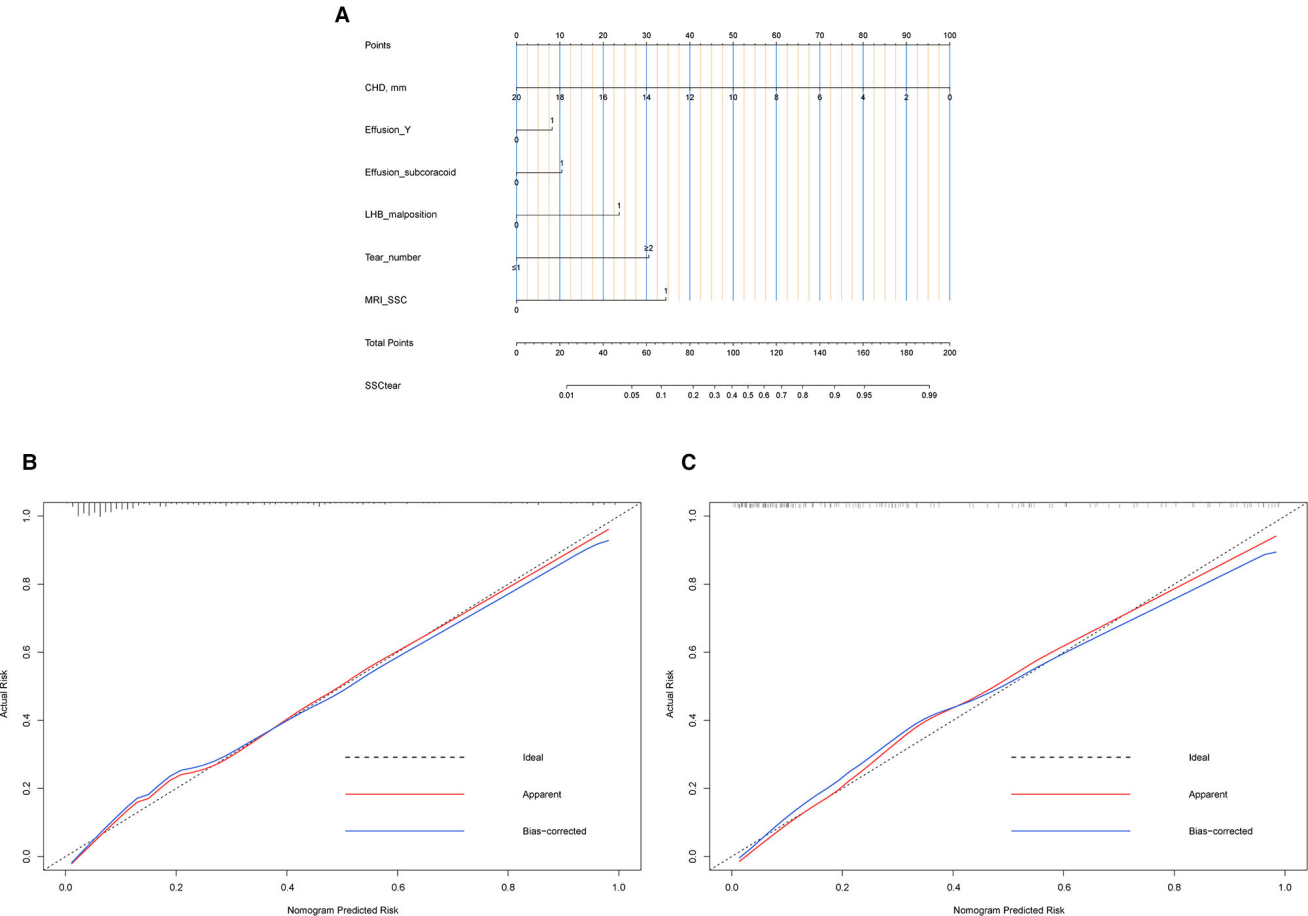
Nomogram and calibration. (**A**) Nomogram. To use the nomogram, find the position of each variable on the corresponding axis, draw a line to the points axis for the number of points, add the points from all of the variables, and draw a line from the total points axis to determine the tear risk of SSC tendon at the lower line of the nomogram. (**B**) Calibration plot of the training data set. Validity of the predictive performance of the nomogram in estimating the risk of SSC tendon tear with the 1,000-sample bootstrapped calibration plot. The calibration plot showed good agreement between predicted and observed outcomes in the training data set. (**C**) Calibration plot of validation data set. The calibration plot showed good agreement between predicted and observed outcomes in the validation data set.

A nomogram to predict SSC tears incorporating these six sensitive predictors is shown in Figure 4. The generated model was internally validated with the 1,000 bootstrap validation method. This nomogram demonstrated a good discriminative ability of the training cohort with a C index of 0.878 (95% CI, 0.839–0.918). In the validation cohort, the nomogram displayed a C index of 0.890 (95% CI, 0.840–0.940) for the risk estimation of SSC tendon tears. Both calibration plots graphically showed good agreement on the risk estimation by the nomograms of these two cohorts (mean absolute error: 0.018 vs. 0.027) (Figure 4). Patients with decreased CHD (oblique sagittal plane), effusion (Y-face), subcoracoid effusion, LHB malposition, multiple PS rotator cuff tears, and considering SSC tendon tears on MRI (direct signs) presented with higher nomogram points which indicated a greater risk of SSC tendon tears.

**Figure 5:**
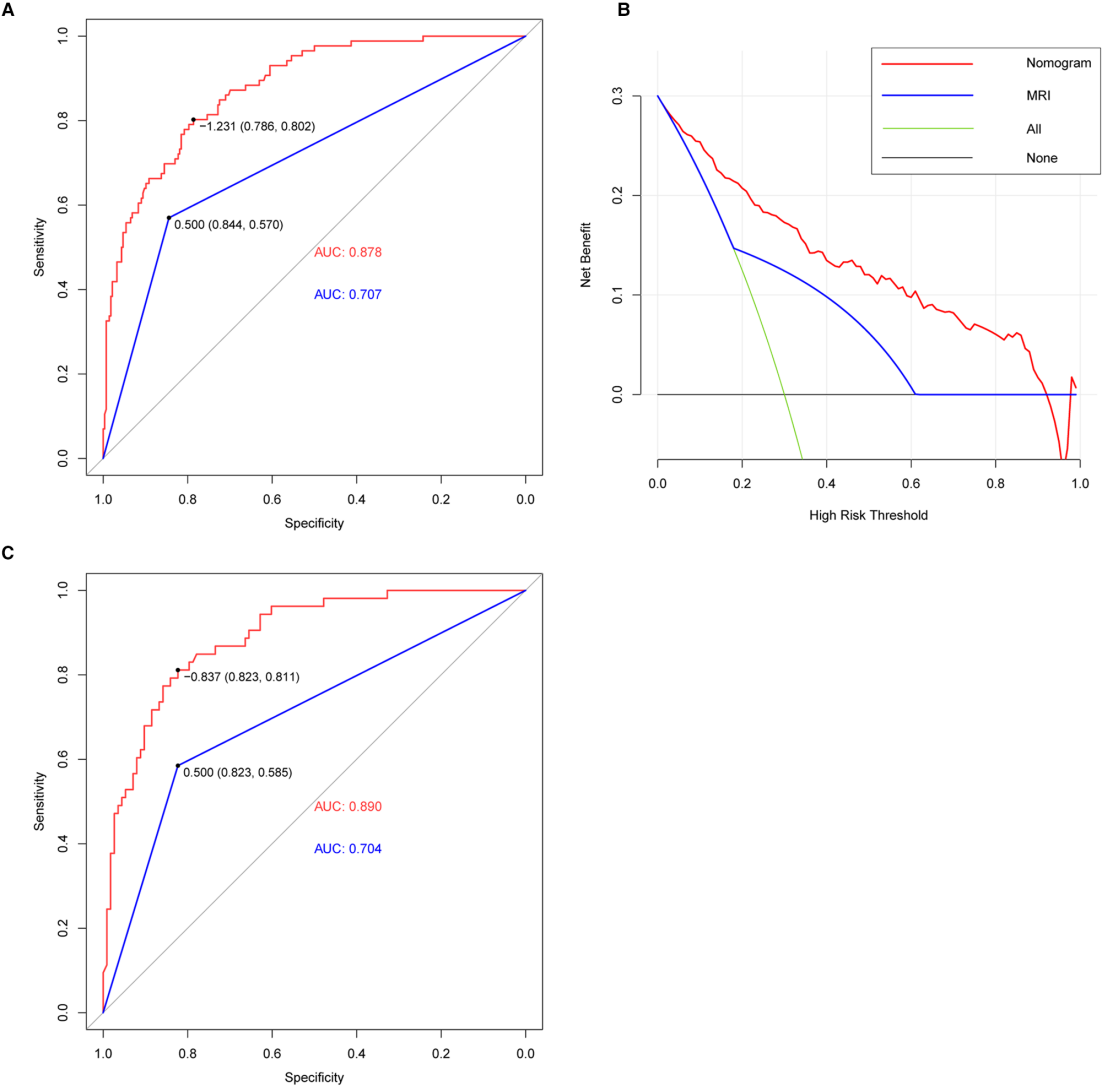
ROC and DCA curve. (**A**) AUC of the nomogram (red line) and MRI (blue line) (nomogram: 0.878, MRI: 0.707) in the training cohort. The nomogram provided better diagnostic performance compared with MRI. (**B**) DCA of nomogram (red line) and MRI (blue line). DCA curve analysis showed that the nomogram had achieved a higher clinical application value and better clinical practicability than MRI. (**C**) AUC of nomogram (red line) and MRI (blue line) (nomogram: 0.890, MRI: 0.704) in the validation cohort. The nomogram also provided better diagnostic performance in the validation cohort.

To compare the diagnostic performance of this nomogram with MRI diagnosis (based on direct signs), the study calculated the sensitivity, specificity, predictive value, and likelihood ratio. Eventually, the sensitivity, specificity, positive predictive value, negative predictive value, positive likelihood ratio, and negative likelihood ratio of these two methods (nomogram versus MRI diagnosis) was 80.2% vs. 57.0%, 78.6% vs. 84.4%, 53.9% vs. 53.3%, 92.7% vs. 86.3%, 3.75 vs. 3.66, and 0.25 vs. 0.51, respectively (Table 2). The area under the receiver-operating characteristic (ROC) curve for these two methods also indicated a superior performance of this nomogram in both training and validation cohorts (Figure 5). We also performed DCA to assess the clinical utility of these two methods (Figure 5). The result suggested that when the threshold probability is between 3% and 93%, the prediction model can yield a good net benefit with higher clinical application value and better clinical practicability than MRI diagnosis based on direct signs.

**Table 2:** Diagnostic performance of nomogram and MRI in SSC tears.

Variable	Value nomogram (95% CI)	Value MRI (95% CI)
Area under ROC curve	0.878 (0.839–0.918)	0.707 (0.639–0.705)
Sensitivity, %	80.2 (70.0–87.7)	57.0 (45.9–67.5)
Specificity, %	78.6 (73.2–83.2)	84.4 (79.5–88.4)
Positive predictive value, %	53.9 (44.9–62.7)	53.3 (42.6–63.6)
Negative predictive value, %	92.7 (88.4–95.6)	86.3 (81.5–90.1)
Positive likelihood ratio	3.75 (2.92–4.81)	3.66 (2.63–5.09)
Negative likelihood ratio	0.25 (0.16–0.39)	0.51 (0.40–0.65)

**Figure 6:**
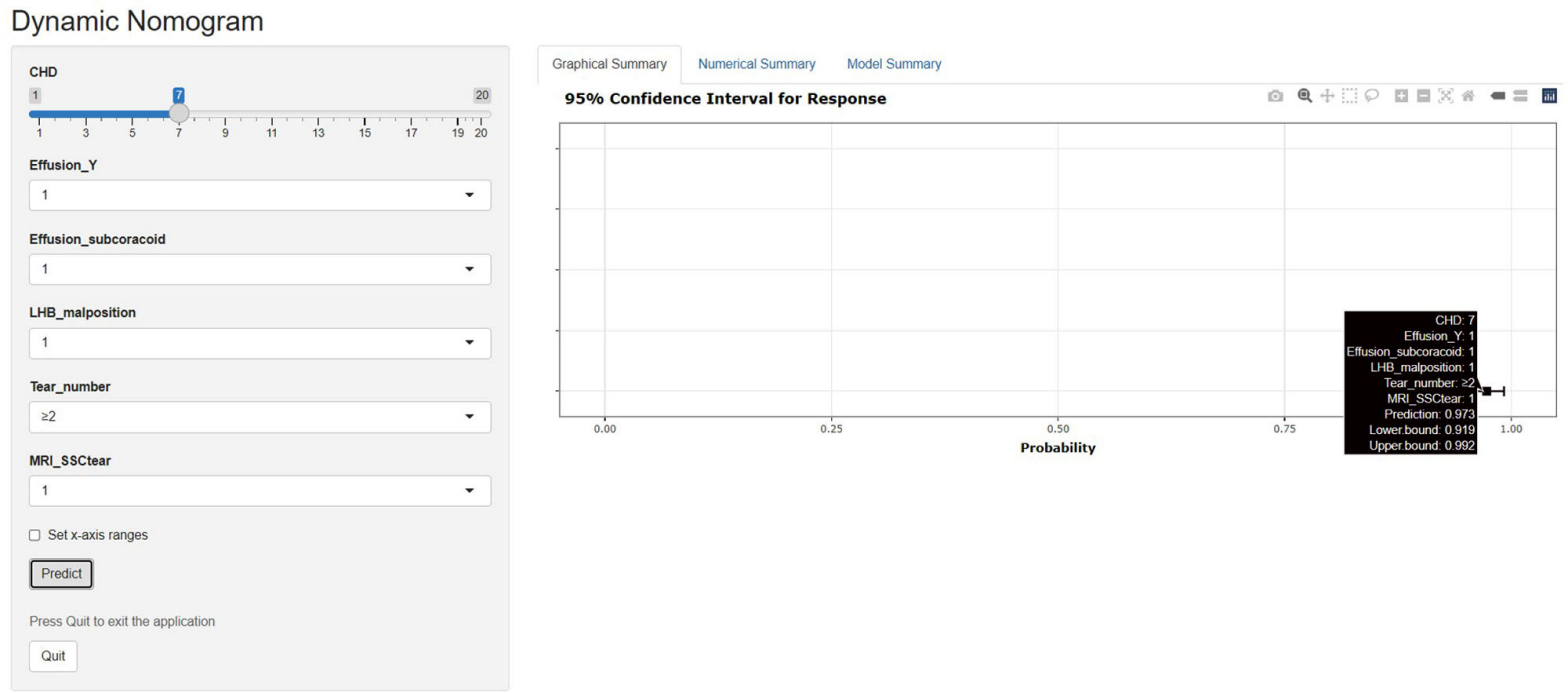
Screenshot of the web-based dynamic nomogram used to predict SSC tendon tears (https://cmuxwn.shinyapps.io/DynNomapp/). This patient combined with effusion (Y-face), subcoracoid effusion, LHB malposition, multiple PS rotator cuff tears, 7 cm of CHD, and considering SSC tendon tears on MRI, and the predicted probability of SSC tendon tear was 97.3% (black line).

Based on the superior nomogram, the study constructed a web-based dynamic probability calculator (https://cmuxwn.shinyapps.io/DynNomapp/) to forecast the probability of SSC tears (Figure 6). It is very advantageous to input the sensitive predictors on the web-based nomogram for real-time individualized prediction of tear probability. The black line represented the probability (97.3%) and 95% CI (0.919–0.992) of SSC tendon tears in a patient with effusion (Y-face), subcoracoid effusion, LHB malposition, multiple PS rotator cuff tears, 7 cm of CHD, and considering SSC tendon tears on MRI (direct signs) (Figure 6).

## Discussion

The ability to identify patients with SSC tendon tears needs to be improved. Considering that the accuracy of MRI diagnosis based on direct signs was not excellent, and doctors could not obtain the likelihood of tear in each patient, we comprehensively evaluated the diagnostic value of these factors and identified highly sensitive predictors. Patients with decreased CHD (oblique sagittal), effusion (Y-face), subcoracoid effusion, LHB malposition, multiple PS rotator cuff tears, and considering SSC tendon tears on MRI (direct signs) were accompanied by a higher risk of SSC tendon tears. We first provided a web-based dynamic nomogram that could provide superior prediction performance to identify SSC tendon tears, even in patients with small (involving an upper third of SSC) or partial tears.

Previous studies implied that the morphology of the coracoid and its relationship with the surrounding structures were closely related to the subscapular tendon tear (20, 21, 29). Some researchers suggested that CHD, CO, and CHI were potential predictors of subscapular tendon tear (20, 21, 30), although others presented different insights (15, 31). To better evaluate the predictive value of CHD in SSC tears, this study measured CHD at the axial and oblique sagittal planes. The results suggested that a decreased CHD (oblique sagittal plane) was highly sensitive to predicting SSC tendon tears, while CHD measured on the axial plane, CHI, and CO were not recommended as sensitive predictors.

Anatomically, the subscapular tendon is closely interdependent with the PS rotator cuff and long head tendon of the biceps (LHB) to assist the formation of the biceps pully structure. Researchers found (17, 32) that the malposition (dislocation/subluxation) of the LHB on MRI could predict a full-thickness tear of the subscapular tendon. Our search demonstrated that LHB malposition was a reliable indicator for predicting SSC tears. Mehta et al. reported that a greater proportion of concomitant full-thickness PS cuff tears could be observed in shoulders with subscapular tendon tears (16). However, our study suggested that the number of PS tendon tears had superior predictive value in diagnosing SSC tendon tears.

Previous studies found that fluid signs around the SSC were associated with SSC tears (18, 23, 25). To better evaluate the predictive value of these fluid signs, we introduced a new index, namely, the fluid area ratio. Although we did not find any correlation of lesser tuberosity cyst with SSC tears, the result pointed out that patients with effusion of Y-face plane and subcoracoid on MRI indicated a higher risk of SSC tendon tears.

Eventually, six indicators were determined as sensitive predictors to assist orthopedic clinicians in diagnosing SSC tendon tears. This study firstly established and validated a nomogram according to clinical and MRI features to predict patients with SSC tears. As a new evaluation tool based on reliable predictors, the nomogram was convenient for providing individualized risk estimation to assist doctors in diagnosis. This dynamic nomogram achieved superior diagnostic performance in both training and validation cohorts, which is helpful in reducing the missed diagnosis rate. Some limitations also exist in our study. This analysis was based on the data of a single institution; it was better to validate the model with multiple institutions in further study. Considering physical examination was susceptible to the subjectivity of patients and clinicians with limited accuracy reported in the previous literature (33), we did not evaluate this factor. As an important diagnostic routine, we recommended that doctors perform a comprehensive diagnostic evaluation in conjunction with physical examinations after obtaining the risk prediction value and imaging diagnosis.

## Conclusion

There are still some challenges in accurately diagnosing SSC tendon tears based on conventional MRI. The study provided a new evaluation tool to assist clinicians in risk estimation and diagnosis. As a good supplementary diagnostic tool, this web-based dynamic nomogram can help doctors better identify SSC tendon tears, even in patients with small or partial tears.

## Data Availability

The raw data supporting the conclusions of this article will be made available by the authors without undue reservation.

## References

[1] Waldt S Bruegel M Mueller D Holzapfel K Imhoff AB Rummeny EJ Rotator cuff tears: assessment with MR arthrography in 275 patients with arthroscopic correlation. Eur Radiol. (2007) 17(2):491–8. 10.1007/s00330-006-0370-7 16969638

[2] Lee JH Yoon YC Jee S Kwon JW Cha JG Yoo JC. Comparison of three-dimensional isotropic and two-dimensional conventional indirect MR arthrography for the diagnosis of rotator cuff tears. Korean J Radiol. (2014) 15(6):771–80. 10.3348/kjr.2014.15.6.771 25469089 PMC4248633

[3] Narasimhan R Shamse K Nash C Dhingra D Kennedy S. Prevalence of subscapularis tears and accuracy of shoulder ultrasound in pre-operative diagnosis. Int Orthop. (2016) 40(5):975–9. 10.1007/s00264-015-3043-9 26585865

[4] Lin L Yan H Xiao J He Z Luo H Cheng X The diagnostic value of magnetic resonance imaging for different types of subscapularis lesions. Knee Surg Sports Traumatol Arthrosc. (2016) 24(7):2252–8. 10.1007/s00167-014-3335-4 25253237

[5] Sela Y Eshed I Shapira S Oran A Vogel G Herman A Rotator cuff tears: correlation between geometric tear patterns on MRI and arthroscopy and pre- and postoperative clinical findings. Acta Radiol. (2015) 56(2):182–9. 10.1177/0284185114520861 24445094

[6] Ward JRN Lotfi N Dias RG McBride TJ. Diagnostic difficulties in the radiological assessment of subscapularis tears. J Orthop. (2018) 15(1):99–101. 10.1016/j.jor.2018.01.016 29657448 PMC5895898

[7] Malavolta EA Assunção JH Gracitelli MEC Yen TK Bordalo-Rodrigues M Ferreira Neto AA. Accuracy of magnetic resonance imaging (MRI) for subscapularis tear: a systematic review and meta-analysis of diagnostic studies. Arch Orthop Trauma Surg. (2019) 139(5):659–67. 10.1007/s00402-018-3095-6 30539284

[8] Banerjee M Müller-Hübenthal J Grimme S Balke M Bouillon B Lefering R Moderate value of non-contrast magnetic resonance imaging after non-dislocating shoulder trauma. Knee Surg Sports Traumatol Arthrosc. (2016) 24(6):1888–95. 10.1007/s00167-014-3102-6 24923686

[9] Ramadan LB Baptista E Souza FF Gracitelli MEC Assunção JH Andrade-Silva FB Diagnostic accuracy of preoperative magnetic resonance imaging for detecting subscapularis tendon tears: a diagnostic test study. Sao Paulo Med J. (2020) 138(4):310–6. 10.1590/1516-3180.2020.014605062020 32844908 PMC9673826

[10] Yoo JC Rhee YG Shin SJ Park YB McGarry MH Jun BJ Subscapularis tendon tear classification based on 3-dimensional anatomic footprint: a cadaveric and prospective clinical observational study. Arthroscopy. (2015) 31(1):19–28. 10.1016/j.arthro.2014.08.015 25442662

[11] Furukawa R Morihara T Arai Y Ito H Kida Y Sukenari T Diagnostic accuracy of magnetic resonance imaging for subscapularis tendon tears using radial-slice magnetic resonance images. J Shoulder Elbow Surg. (2014) 23(11):e283–90. 10.1016/j.jse.2014.03.011 24927884

[12] Cigolotti A Biz C Lerjefors E de Iudicibus G Belluzzi E Ruggieri P. Medium- to long-term clinical and functional outcomes of isolated and combined subscapularis tears repaired arthroscopically. Arch Med Sci. (2021) 17(5):1351–64. 10.5114/aoms.2020.97714 34522265 PMC8425253

[13] Yoon TH Kim SJ Choi YR Keum HS Chun YM. Clinical outcomes for isolated SSC tears with advanced fatty infiltration: nonoperative treatment versus arthroscopic single-row repair. Orthop J Sports Med. (2021) 9(2):2325967120975754. 10.1177/2325967120975754 33614807 PMC7869153

[14] Ryu HY Song SY Yoo JC Yun JY Yoon YC. Accuracy of sagittal oblique view in preoperative indirect magnetic resonance arthrography for diagnosis of tears involving the upper third of the subscapularis tendon. J Shoulder Elbow Surg. (2016) 25(12):1944–53. 10.1016/j.jse.2016.02.038 27282733

[15] Cetinkaya M Ataoglu MB Ozer M Ayanoglu T Kanatli U. Subscapularis tendon slip number and coracoid overlap are more related parameters for subcoracoid impingement in subscapularis tears: a magnetic resonance imaging comparison study. Arthroscopy. (2017) 33(4):734–42. 10.1016/j.arthro.2016.09.003 27939068

[16] Mehta SK Teefey SA Middleton W Steger-May K Sefko JA Keener JD. Prevalence and risk factors for development of subscapularis and biceps pathology in shoulders with degenerative rotator cuff disease: a prospective cohort evaluation. J Shoulder Elbow Surg. (2020) 29(3):451–8. 10.1016/j.jse.2019.11.012 32067709 PMC7178076

[17] Yoon JS Kim SJ Choi YR Lee W Kim SH Chun YM. Medial subluxation or dislocation of the biceps on magnetic resonance arthrography is reliably correlated with concurrent SSC full-thickness tears confirmed arthroscopically. BioMed Res Int. (2018) 2018:2674061. 10.1155/2018/2674061 30271779 PMC6151251

[18] Cetinkaya M Öner AY Ataoglu MB Ozer M Ayanoglu T Kanatli U. Lesser tuberosity cysts and their relationship with subscapularis tears and subcoracoid impingement. J Orthop Sci. (2017) 22(1):63–8. 10.1016/j.jos.2016.09.018 27769600

[19] Lafosse L Jost B Reiland Y Audebert S Toussaint B Gobezie R. Structural integrity and clinical outcomes after arthroscopic repair of isolated subscapularis tears. J Bone Joint Surg Am. (2007) 89(6):1184–93. 10.2106/00004623-200706000-00005 17545420

[20] Leite MJ Sá MC Lopes MJ Matos RM Sousa AN Torres JM. Coracohumeral distance and coracoid overlap as predictors of subscapularis and long head of the biceps injuries. J Shoulder Elbow Surg. (2019) 28(9):1723–7. 10.1016/j.jse.2019.01.012 31014558

[21] Zhang H Zhang Q Li ZL. Coracohumeral index and coracoglenoid inclination as predictors for different types of degenerative subscapularis tendon tears. Int Orthop. (2019) 43(8):1909–16. 10.1007/s00264-018-4078-5 30159801

[22] Meyer DC Zimmermann SM Wieser K Bensler S Gerber C Germann M. Lengthening of the subscapularis tendon as a sign of partial tearing in continuity. J Shoulder Elbow Surg. (2016) 25(1):31–7. 10.1016/j.jse.2015.06.014 26234662

[23] Shim JW Pang CH Min SK Jeong JY Yoo JC. A novel diagnostic method to predict subscapularis tendon tear with sagittal oblique view magnetic resonance imaging. Knee Surg Sports Traumatol Arthrosc. (2019) 27(1):277–88. 10.1007/s00167-018-5203-0 30317525

[24] Wissman RD Ingalls J Hendry D Gorman D Kenter K. Cysts within and adjacent to the lesser tuberosity: correlation with shoulder arthroscopy. Skeletal Radiol. (2012) 41(9):1105–10. 10.1007/s00256-012-1366-9 22286591

[25] Turkmen I Altun G Celik H Bilsel K. Can subcoracoid cyst formation be a sign of anterosuperior rotator cuff tears and biceps pulley lesions? a prospective radiologic and arthroscopic correlation study. J Shoulder Elbow Surg. (2020) 29(8):1665–70. 10.1016/j.jse.2019.11.036 32192879

[26] Chillemi C Paglialunga C Guerrisi M Mantovani M Osimani M. Arthroscopic transosseous repair of rotator cuff tear and greater tuberosity cysts. Arthrosc Sports Med Rehabil. (2020) 2(3):e241–e50. 10.1016/j.asmr.2020.03.005 32548590 PMC7283963

[27] Chin K Chowdhury A Leivadiotou D Marmery H Ahrens PM. The accuracy of plain radiographs in diagnosing degenerate rotator cuff disease. Shoulder Elbow. (2019) 11(1 Suppl):46–51. 10.1177/1758573217743942 31019562 PMC6463379

[28] Sauerbrei W Royston P Binder H. Selection of important variables and determination of functional form for continuous predictors in multivariable model building. Stat Med. (2007) 26(30):5512–28. 10.1002/sim.3148 18058845

[29] Hodax JD Shah KN Campbell SE Cameron KL Owens BD. Measurement of the coracohumeral distance on magnetic resonance imaging in a large patient cohort. J Shoulder Elbow Surg. (2021) 30(2):408–12. 10.1016/j.jse.2020.05.032 32561480

[30] Zhu S Tan J Wu D Hu N Huang W Chen H. Bilateral coracohumeral distance discrepancy is associated with subscapularis tear in rotator cuff rupture patients. Knee Surg Sports Traumatol Arthrosc. (2021) 29(12):3936–42. 10.1007/s00167-021-06597-6 33956166

[31] Tollemar VC Wang J Koh JL Lee MJ Shi LL. Coracoid morphology is not associated with subscapularis tears. J Shoulder Elbow Surg. (2020) 29(6):1162–7. 10.1016/j.jse.2019.11.008 32057656

[32] Kim BR Lee J Ahn JM Kang Y Lee E Lee JW Predicting the clinically significant subscapularis tendon tear: malposition and tear of the long head of the biceps tendon on shoulder magnetic resonance imaging. Acta Radiol. (2021) 62(12):1648–56. 10.1177/0284185120980017 33325726

[33] Gismervik S Drogset JO Granviken F Rø M Leivseth G. Physical examination tests of the shoulder: a systematic review and meta-analysis of diagnostic test performance. BMC Musculoskelet Disord. (2017) 18(1):41. 10.1186/s12891-017-1400-0 28122541 PMC5267375

